# On the Treatment of Acute Inflammations of the Middle Ear

**Published:** 1889-03

**Authors:** Charles F. Pickering


### Clinical Record.

---

### ON THE TREATMENT OF ACUTE INFLAMMATIONS OF THE MIDDLE EAR. By CHARLES F. PICKERING, F.R.C.S.

Acute inflammations of the middle ear are of two kinds: (1) Those which are not accompanied by suppuration, and (2) Those in which suppuration takes place.

These inflammations may be brought about by zymotic diseases, by injury, or cold; and should suppuration take place, the complications arising during its existence are often so serious that it is of the greatest importance to consider what is the best method of treatment for preventing, or (if discharge takes place) for arresting, the formation of pus. To one like myself, having the charge at a hospital of a special department for the treatment of diseases of the ear, the ravages one sees connected with these affections are peculiarly distressing, especially when one feels that by an early and proper method of treatment, they may be avoided.

The first warning symptom of commencing acute inflammation of the middle ear is pain, and usually acute pain—sometimes so acute and distracting, that the patient may be said, without exaggeration, to go "mad with pain:" although in some few cases there is no pain at all; of these I have seen only a few examples.

When the ear is examined, and the surgeon finds that an acute inflammation of the tympanic cavity exists, he should immediately take measures to thoroughly purify the external meatus, so that, should suppuration unfortunately become established, the pus is discharged into an aseptic cavity, which, being occluded by a plug of antiseptic wool, prevents the intrusion of germs.

Means should also be at once adopted to arrest, if possible, the course of the disease. Leeching behind, and more especially in front of the ear, will often instantly stop the pain, and lead to a subsidence of the trouble: next, fomenting the ear with hot water at short intervals, with a large sponge, is desirable. Poultices are most objectionable, as they favour suppuration and are dirty. Extract of belladonna and glycerine in equal parts should be smeared around the ear, or chloroform and opium liniments should be applied; but nothing should be poured into the meatus that is not perfectly aseptic. Instillations of cocaine in a warm saturated solution of boracic acid of 20 per cent. strength, or of plain hot boracic lotion, may be used. The affected side of the head should be covered by cotton-wool. Medicines given internally should be opiates to ease pain, and other remedies suggested by the constitutional state of the patient. Antimony (if the patient's condition allows of it) may be tried, and will often quickly lead to a subsidence of the trouble. The primary purification of the ear is best carried out by syringing with warm perchloride lotion, 1 — 2000; after which a little iodoform should be placed in the meatus, which is lastly plugged with salicylate wool.

Should suppuration occur, the discharges should be cleared from the ear by daily syringing the new aseptic meatus with warm boracic acid lotion, and the wool plug which forms the antiseptic dressing always replaced.

Under this perfect method of antiseptic treatment, the suppuration is, in most cases, arrested in a few days, and perforations of the drum rapidly heal, even if very large, and usually leave the hearing unimpaired.

Air should never be forced into the tympanic cavity during the acute stage, or, if pus is formed, before the abscess bursts; it will almost invariably lead to an increase of the symptoms.

After the discharge has lasted a day or two, air should be injected up the Eustachian tube, so as to clear the tympanum of pus and free the tube from obstruction—which is now easily done, the swelling having subsided. As the air is, I believe, filtered in its passage up the tube and so freed from germs, no harm can arise from that source by inflation. Pulv. acid. boracic. may be insufflated into the external meatus after each syringing; and if the discharge has any tendency to become chronic, pulv. aluminis should be mixed with the boracic powder. If this treatment were carried out in all cases, we should see very little chronic suppuration of the ear.

I am not in favour of opening the tympanic cavity by incising the drum when pus is suspected, except under very special circumstances. Opening an abscess of the middle ear seems a tempting surgical procedure, but practically it is not often needed; nature makes the best opening, and all cases do well if properly treated afterwards. If an opening is made, it is often a very imperfect means of exit for the pus, and nature has to do the work herself by making an opening somewhere else; but I think an incision is advisable when there is severe constitutional disturbance, or prolonged great pain, or when the abscess is slow in perforating the drum.

Acute inflammations of the middle ear usually stop short of suppuration, and quickly subside under the treatment recommended for arresting the course of the disease, the hearing also becoming quite normal; but should the hearing be left impaired, it will be improved by inflations of the tympanic cavity, which are best done with Politzer's bag. If much deafness remains fluid exudations probably exist in the middle ear, and if they cannot be removed by inflations, accompanied by placing the head in a position to allow the fluid to run down the Eustachian tube by the force of gravity, the drum must be punctured and the fluid expelled into the meatus, and so removed. Where the hearing is slow in recovering, injections into the tympanic cavity may be used through the tympanic catheter. These should be astringent, if slight exudations exist; or, if there is thickening of the mucous membrane, solutions of pot. iodid. or vapour of iodine are most useful. These, shortly, are the means I employ, and which I have seldom to supplement by other remedies.

